# Fruit-specific overexpression of lipoyl synthase increases both bound and unbound lipoic acid and alters the metabolome of tomato fruits

**DOI:** 10.3389/fpls.2025.1545011

**Published:** 2025-05-19

**Authors:** María Paz Covarrubias, Felipe Uribe, Daniela Arias-G, Pamela Cabedo, Michael Handford

**Affiliations:** Centro de Biología Molecular Vegetal, Department of Biology, Faculty of Sciences, University of Chile, Santiago, Chile

**Keywords:** antioxidant, lipoylation, polygalacturonase promoter, *Solanum lycopersicum*, TCA cycle

## Abstract

**Introduction:**

Lipoic acid (LA) is a key, yet overlooked player in primary metabolism, due to its role as a cofactor for various multi enzymatic complexes such as the E2 subunits of pyruvate dehydrogenase (PDH) and alpha-ketoglutarate dehydrogenase (kGDH). In recent years, this molecule has seen renewed interest given its strong antioxidant properties and its applications as a dietary supplement. The mechanisms behind the synthesis of LA in vivo have been elucidated, identifying lipoyl synthase (LIP1) as the key enzyme required for this process.

**Methods:**

Therefore, in this work, we used the fruit-specific polygalacturonase (PG) promoter to guide *Solanum lycopersicum *(tomato) *LIP1* (*SlLIP1*) overexpression in stably transformed tomatoes.

**Results:**

The resulting plants presented higher transcript levels of *SlLIP1* in a fruit-specific manner, accumulated more bound and unbound LA yet lacked major phenotypic defects at both the vegetative and reproductive growth stages. Furthermore, changes in the expression of genes related to LA synthesis were explored and a metabolomic study was carried out. Specific metabolite patterns were clearly distinguishable between untransformed and stably transformed lines. For instance, trehalose 6-phosphate, GABA and proline levels were generally higher, whilst glucose 6-phosphate and UDP-glucose levels were lower in fruits of the *SlLIP1* transformants.

**Discussion:**

In addition, as the overexpression of *SlLIP1* results in lower transcript levels of *E2 PDH* and *E2 kGDH*, and enhanced amounts of LA-bound targets, we speculate that the proportion of unlipoylated E2 subunits of PDH and kGDH may have decreased. This work could assist in obtaining crops with a higher LA content and therefore improved health benefits.

## 1 Introduction

Antioxidants are molecules known generally for their ability to provide reducing power and neutralize reactive oxygen species (ROS; Turrens, 2003). Although ROS are a natural byproduct of metabolism, they are often produced in electron transport chains, and even act as signaling molecules; excessive ROS production is also associated with cellular damage in organisms and aging (Turrens, 2003). Because of this, research into antioxidant molecules has surged in recent years for their applications in many industries, including in the food, pharmaceutical, agronomical, and cosmetic sectors, among others (Jimenez-Lopez et al., 2020; Lupo, 2001; Neha et al., 2019; Shahidi, 2000; Sun et al., 2024).

Lipoic acid (LA) and its reduced form (dihydrolipoic acid, DHLA) are antioxidants with several unique properties. LA is an eight-carbon molecule characterized by its two thiol groups in the carbon six and carbon eight positions that are responsible for its antioxidant nature. Like many antioxidants, LA is capable of neutralizing a wide range of ROS, regenerating other antioxidant molecules such as glutathione, and chelating heavy metals (Gora̧ca et al., 2011; Matsugo et al., 1996; Packer et al., 1995; Scott et al., 1994). However, unlike other antioxidants, LA is both water and lipid soluble in its free form (Packer et al., 1995). For these reasons, LA has been the focus of many studies that have demonstrated its beneficial influence over human health, such as preventing the effects of diabetic neuropathies, and stabilizing cognitive functions in Alzheimer’s patients, or in other neurodegenerative diseases induced by oxidative stress (Borcea et al., 1999; Gilgun-Sherki et al., 2001; Packer et al., 2001). Other organisms, including plants, have also been shown to benefit from applications of LA. For example, exogenous LA ameliorates the effects of oxidative stress caused by high salinity in wheat seedlings, and promotes water-deficit tolerance in maize (Gorcek and Erdal, 2015; Saruhan Guler et al., 2021).

An additional feature of LA is that it can be found as an essential cofactor. In this role, LA is necessary for the functioning of various enzymatic complexes that participate in primary and secondary metabolism. These lipoylated targets include the E2 subunits of pyruvate dehydrogenase (PDH), alpha-ketoglutarate dehydrogenase (kGDH), and branched-chain 2-oxoacid dehydrogenase (BCDH), as well as the H-protein of the glycine decarboxylase complex (GDC) (Harmer et al., 2014). In plants, LA can be added onto its cognate enzyme through two distinct pathways: the direct ligation of an exogenous LA molecule onto a cognate enzyme in a ‘salvage’ pathway via lipoate protein ligase (LplA), or through the assembly of a lipoate cofactor *de novo*, from an octanoyl precursor followed by the addition of two sulfur atoms by lipoyl synthase (LIP1) (Ewald et al., 2014a; Yasuno and Wada, 2002). In the latter case, the octanoyl precursor is bound firstly to an acyl carrier protein (ACP) by octanoyltransferase (LIP2), enabling the 8-carbon precursor to be shuttled to either a E2 subunit (PDH, kGDH, BCDH) or H-protein (GDC) before being thiolyated by LIP1 in a rare example of a cofactor being assembled *in situ* (Ewald et al., 2014a; Sarvananda et al., 2023). Therefore, both the unbound (LA) and bound (lipoylated proteins) forms, acting as an antioxidant and cofactor, respectively, require the addition of thiol groups by LIP1, making it the key step in the metabolism of this molecule (Ewald et al., 2014).

It is known that the E2 subunit of PDH is a target for lipoylation and there are two PDH complexes in plants, with one being present in mitochondria (PDHm) and participating in the TCA cycle, while the other is found in chloroplasts (PDHc) and plays a role in *de novo* lipid biosynthesis (Tovar-Méndez et al., 2003). Studies of plastidial sunflower (LIP1p; *Helianthus annuum* L.) lipoyl synthase in *Arabidopsis* revealed that expression of this gene results in a redistribution of the glycerolipid species being produced, attributed to the depletion of S-adenosylmethionine (SAM or Ado-Met), whose reductive cleavage by LIP1 is required for thioylation to take place (Harmer et al., 2014; Martins-Noguerol et al., 2020). SAM also participates in various other cellular processes, including as a precursor for ethylene synthesis, a phytohormone associated with senescence and stress responses, as well as regulatory processes such as DNA methylation (Cronan, 2014; Harmer et al., 2014; Lu, 2000; Sauter et al., 2013). Indeed, expression of cotton (*Gossypium hirsutum* L.) LIP1 delayed senescence in *Arabidopsis* (Chen et al., 2021). Because LA is an antioxidant and participates in a central role by lipoylating several enzymatic complexes, it is expected that modifications in LA metabolism would lead to widespread metabolic changes.

In this work, we generated a system that allowed us to examine the effects of the organ-specific over-expression of LIP1. To do so, we overexpressed mitochondrial LIP1 from *Solanum lycopersicum* (tomato; *SlLIP1*, Solyc07g054540; Araya-Flores et al., 2020) in a fruit-specific manner in stably-transformed tomato. This was achieved by guiding the expression of *SlLIP1* with the promoter region of the tomato *polygalacturonase* gene (*PG*) which is triggered during fruit ripening in normal development (Montgomery et al., 1993). PG is a cell-wall degrading enzyme, and publicly-available transcriptome data (bar.utoronto.ca/eplant_tomato/) demonstrates that *PG* expression is detected exclusively in maturing tomato fruits. Changes in phenotype, gene expression, protein accumulation, and the fruit metabolome were assessed to elucidate the global impact of altered LA metabolism in plants, and especially in fruits.

## 2 Materials and methods

### 2.1 Vector construction

**Figure 1:**
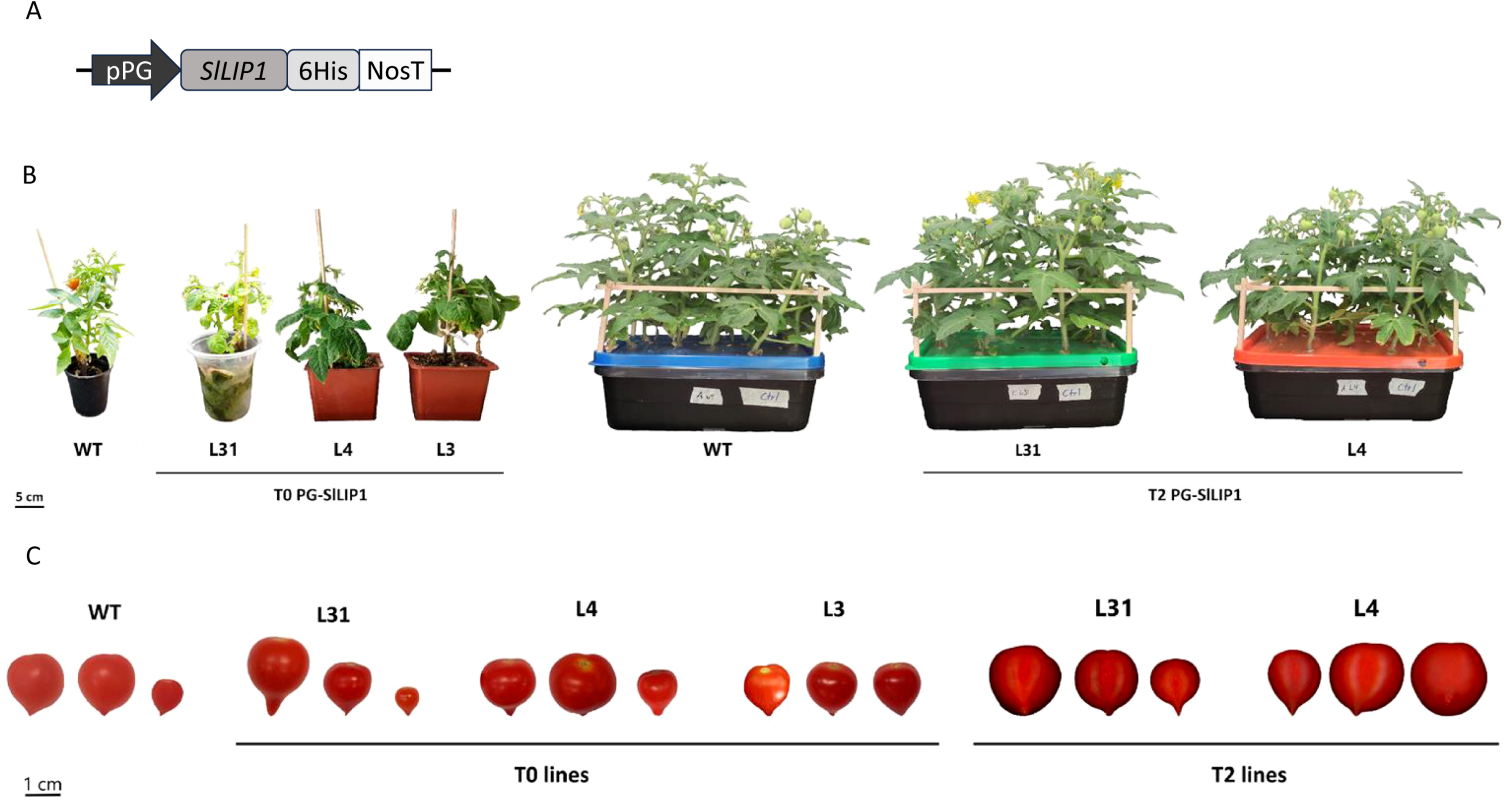
Generation of tomato plants overexpressing *SlLIP1.* **(A)** Diagram of the pCP vector used to transform tomato cv 'Micro-Tom'. **(B)** Three independent T0 lines (4-months old, L31, L4 and L3) and two in the T2 generation (3-months old, L31 and L4) exhibited normal development compared to WT. **(C)** Three representative fruits of PG-SlLIP1 lines at T0 and T2 generations.

The full-length CDS of *SlLIP1* was previously cloned into the pGWB8 binary vector (Araya-Flores et al., 2020). Using primers containing the *Asc*I restriction site (primer pair 1-2; **Supplementary Table 1**), *SlLIP1* was amplified from the ATG start codon to the stop codon of the vector, including a 6xHis tag, and subcloned into the pCR™8/GW/TOPO^®^ TA (Invitrogen), generating the pCR8-*SlLIP1* construct. The pCP binary vector is based on the backbone of the pB7FWG2-AtDXR vector, containing a 7,854 bp fragment with the T-DNA right and left borders (RB and LB) and the BAR gene, which confers resistance to BASTA^®^ or glufosinate (herbicide) in plants (Arias et al., 2021). This pCP vector already contained an 804 bp polygalacturonase (PG) promoter fragment driving the transcription of the *DcLCYB* gene. To excise *DcLCYB*, 1 μg of pCP was digested with 10 U *Asc*I (New England Biolabs), following the manufacturer’s protocol, generating linearized pCP with the PG promoter. The same digestion was performed on the pCR8-*SlLIP1* construct to obtain the *SlLIP1* insert. Finally, the *SlLIP1* insert was ligated overnight at 4°C into linearized pCP using 3 U of T4 ligase (Promega) at a 1:1 vector-to-insert ratio, resulting in the PG-*SlLIP1* vector (**Figure 1A**). This final vector was verified by sequencing (Macrogen Co.) and transformed into *Agrobacterium tumefaciens* (EHA105 strain). Positive clones were selected and used for the stable transformation of *Solanum lycopersicum* var. ‘Micro-Tom’.

### 2.2 Tomato stable transformation and *in vitro* culture

*S. lycopersicum* var. ‘Micro-Tom’ was cultivated *in vitro* on solid Murashige & Skoog (MS) medium (4.4% MS salts with vitamins, 1.5% sucrose, and 1.5% agar, pH 5.8) and transformed according to Cruz-Mendívil et al. (2011). Briefly, young leaves from three-week-old plants were excised, and the tips were removed with a sterile scalpel. These explants were incubated in co-culture medium (4.4% MS salts with vitamins, 3% sucrose, 100 μM acetosyringone, 0.1 μM naphthaleneacetic acid, 2 μg/mL trans-zeatin) for 3–4 days before transformation. The explants were then immersed in an *Agrobacterium* culture harboring pCP-*SlLIP1* for 20 min, transferred to fresh co-culture medium, and incubated for 2 days at 23°C in darkness.

Next, the explants were transferred to induction medium (4.4% MS salts with vitamins, 3% sucrose, 2 μg/mL trans-zeatin, 0.1 μg/mL indole-3-butyric acid [IBA], 400 μg/mL timentin, and 0.25 μg/mL BASTA^®^) for callus induction and initial shoot development, which typically takes 4 to 6 weeks. The emerging shoots were then transferred to elongation medium (4.4% MS salts with vitamins, 1.5% sucrose, 400 μg/mL timentin, and 0.35 μg/mL BASTA^®^). Once the shoots reached a height of 8 cm, they were transferred to rooting medium (2.2% MS salts with vitamins, 1.5% sucrose, 5 μg/mL IBA, 400 μg/mL timentin, and 0.5 μg/mL BASTA^®^) until roots formed.

The plants were then acclimated in a greenhouse (16 h light/8 h dark photoperiod with white fluorescent light at 150 µmol/m²/s, 22–25°C) in plastic pots (20 x 10 cm) containing a mix of soil and vermiculite (2:1) or rock wool soaked with hydroponic medium (1.25 mM KNO_3_, 1.5 mM Ca(NO_3_)_2_, 0.75 mM MgSO_4_, 0.5 mM KH_2_PO_4_, 50 µM H_3_BO_3_, 10 µM MnSO_4_, 2 µM ZnSO_4_, 1.5 µM CuSO_4_, 0.075 µM(NH_4_)_6_Mo_7_O_24_, 0.1 mM Na_2_O_3_Si, iron diethylenetriamine pentaacetate, pH 6).

To confirm the insertion of PG-*SlLIP1* into the plant genome, PCR was performed on DNA extracted from leaves using primers designed to amplify the region from the PG promoter to the 6xHis tag (primer pair 3-4, **Supplementary Table 1**).

### 2.3 RNA extraction and quantitative expression analysis

RNA was isolated from fully-expanded leaves and transgenic and wild-type (WT) tomato fruits at the green, breaker, pink and mature red stages (CFR, 1991) using a modified CTAB method (Arias et al., 2021, 2022), and cDNA synthesis was carried out using the ImProm-II™ Reverse Transcription System (Promega), as described in Arias et al. (2021). Quantitative expression analysis (qRT-PCR) was conducted using a Stratagene MX300P system (Agilent Technologies) with Forget-Me-Not™ qPCR Master Mix containing ROX (Biotium). From fruits, the primers used to analyze transcript levels of *SlLIP1* (primer pair 5-6), *SlLIP1p* (primer pair 7-8), *SlLIP2* (primer pair 9-10), *SlLplA* (primer pair 11-12), *E2-PDHm* (primer pair 13-14), *E2-PDHc* (primer pair 15-16), *E2-kGDH* (primer pair 17-18), and *SlSAMS1* (primer pair 19-20) are listed in **Supplementary Table 1**. *Actin7* (primer pair 21-22, **Supplementary Table 1**) was selected as the reference gene (Bokhale et al., 2023). To analyze the introduced *SlLIP1* in leaves, RT-PCR was carried out using primers specific to *SlLIP1* and the 6xHis tag (primer pair 5-4) and *GAPDH* was used as a housekeeping gene (primer pair 23-24). Each qRT-PCR was performed with three biological replicates and two technical replicates. Quantification was calculated using the method described by Pfaffl (2001).

### 2.4 Analysis of lipoic acid bound to proteins

Protein extraction from mature red transgenic and WT tomato fruits was performed according to Nilo-Poyanco et al. (2013). Protein concentrations were determined using the BCA method (Thermo Scientific), and samples were loaded onto an SDS-PAGE gel to confirm protein integrity via Coomassie Blue staining. Subsequently, immunoblot analysis was conducted using an anti-LA antibody (1:3000, Calbiochem 437695) and an anti-actin antibody (1:2000, Sigma-Aldrich SAB4301137). Both membranes were incubated with a secondary antibody conjugated to HRP (1:80000, Biorad 170-6515) and visualized using SuperSignal™ West Femto (Thermo Scientific). Signals were captured using a UVITEC Cambridge (Biomolecular Imaging-Alliance ^©^ Software) photodocumentation system, and images were processed using ImageJ software.

For the anti-LA immunoblot, the total signal intensity of each lane was recorded, while for the anti-actin immunoblot, the 42 kDa band was quantified (Jimenez-Lopez et al., 2014). Protein-bound LA quantification was calculated as the ratio between the sum of pixel intensity per area from the LA immunoblot and the pixel intensity per area from the 42 kDa band of the actin immunoblot for each sample, using 3 (T0 and T2) and 5 (WT) replicates. The final result for each transgenic line was normalized relative to the WT sample.

### 2.5 Analysis of unbound lipoic acid

To extract hydrophilic antioxidants, 10 mg of dry weight (DW) from mature red transgenic and WT tomato samples (B+15 days approximately) were ground using liquid nitrogen with a pestle and mortar. Then, 250 μL 0.5% acetic acid in methanol were added, followed by vortexing and sonication for 1 hour. The samples were centrifuged at 7,500 g for 10 min, and the supernatants were freeze-dried. The resulting pellet was resuspended in 10 μL of water to obtain the antioxidant solution.

To detect unbound LA, 1 μL of the antioxidant solution was loaded onto a nitrocellulose membrane, and a dot blot assay was performed. The membrane was blocked with 5% non-fat milk in TBS-T for 30 min. After three washes with TBS-T, the membrane was incubated with an anti-LA antibody (1:3000, Calbiochem 437695) for 30 min, followed by three additional washes with TBS-T. A secondary antibody conjugated to HRP (1:20000, Biorad 170-6515) was then applied, and signals were detected as described in section 2.4. As a positive control, 2 μL of total protein from the T0 PG-SlLIP1 L4 line was used.

### 2.6 Targeted metabolomic analysis

Polar metabolite extraction was performed following the protocol described by Alonso et al. (2010). Briefly, 10 mg DW from mature red transgenic and WT tomato samples were ground using liquid nitrogen with a pestle and mortar. As internal standards, 5 mM ^13^C-glycine, 20 mM ^13^C-mannose, and 1 mM ^13^C-fumarate were added to each tube. Polar metabolites were extracted using 1 mL of boiling water and then incubated in a water bath at over 90°C for 10 min. The extracts were then placed on ice and centrifuged at 17,000 g for 5 min at 4°C. The supernatants were passed through a 0.22 μm filter, and the remaining pellets were subjected to a second extraction. Finally, the water-soluble metabolites were freeze-dried overnight.

The metabolites were resuspended in 500 μL of water, vortexed, and 150 μL were loaded onto a 0.2 μm nanosep MF centrifugal device and centrifuged at 17,000 g for 10 min at 4°C to detect sugars and sugar alcohols. The remaining volume was loaded onto a 3 kDa Amicon Ultra column, centrifuged at 14,000 g for 75 min at 4°C, and kept on ice until injection into the LC-MS/MS.

LC-MS/MS quantification was performed following the method described by Cocuron et al. (2014) to determine sugars, sugar alcohols, amino acids, phosphorylated compounds, and organic acids.

### 2.7 Statistical analysis

Transcript, bound and unbound LA, and metabolite levels were analyzed using a t-test (p < 0.05), with WT serving as the reference parameter for comparison.

Metabolomic data were uploaded to MetaboAnalyst 6.0 (Pang et al., 2024) for statistical analysis (one-factor), including PCA, heatmap, and pathway enrichment analysis on normalized data. Metabolites were used as predictor variables, while genotype (WT and transgenic lines) was used as the response variable. For clustering analysis, the Euclidean distance similarity measure and Ward’s clustering algorithm were applied.

## 3 Results

### 3.1 Stable transformation of tomato with PG-SlLIP1 reduces seed production

Considering the essential role of lipoylation in several enzymes in primary metabolism, the overexpression of lipoyl synthase, the key enzyme required for this process, may be expected to generate pleiotropic phenotypic effects, even if limited to specific organs in plants. Therefore, to increase the LA content in fruits, we performed a stable transformation of tomato cv ‘Micro-Tom’ plants with *SlLIP1* (Solyc07g054540), which encodes mitochondrial lipoyl synthase (Araya-Flores et al., 2020). The CDS of *SlLIP1* was cloned into the pCP vector (Arias et al., 2021), a binary vector that contains an 804 bp fragment upstream of the transcription start site of the *Polygalacturonase* (*SlPG14*, Solyc10g080210.1.1) promoter from *S. chilense* (tomatillo). The expression of *PG* in tomato cv ‘Micro-Tom’ is fruit-specific starting in breaker and peaking at the pink stage (**Supplementary Figure 1A**). pCP also harbors a Nopaline synthase Terminator (NosT), and the *bar* gene, which confers BASTA^®^ resistance. The *SlLIP1* CDS was cloned with a 6xHis tag at the 3’ end (**Figure 1A**).

Using *Agrobacterium*-mediated transformation, young leaves were transformed with pCP harboring PG-SlLIP1, and the regeneration of the explants was carried out *in vitro*. Upon root elongation, plants were transferred *ex vitro* until the appearance of fruits. Three T0 transgenic lines, referred to as L31, L4 and L3, were analyzed and compared to WT plants (**Figure 1B**). By monitoring the growth and development of all lines, it was determined that the height of the aerial part of all plants at the end of the third month of acclimatization (4 months post-transformation) in the greenhouse was similar between L31, L4 and L3 and WT, indicating no significant impact on overall vegetative performance (**Table 1**). When the fruits were analyzed, the main difference observed was in the number of seeds; while WT fruits had seeds in all fruits, a reduction was found in L31 and L4, and no seeds were present in L3 (**Table 1**, **Figure 1C**). Due to this feature, transgenic lines L31 and L4 were propagated until the T2 generation, for further evaluation.

**Table 1:** Phenotypic description of WT and T0 and T2 PG-SlLIP1 transgenic tomato lines.

		Plant phenotype		Fruit phenotype	
	Genotype	Plant height (cm)	Flowering time (d)	Diameter (mm)	Presence of seeds^c^
	WT	18.8	58	13.3 ± 3.8^b^	++
T0 PG-SlLIP1	L31	16.5	40	15.7 ± 2.9^b^	+
	L4	10	67	16.3 ± 2.5^b^	+
	L3	11.5	67	13.0 ± 1.0^b^	–
T2 PG-SlLIP1	L31	22.3^a^	67	18.1 ± 2.3^b^	+
	L4	18.6^a^	60	17.8 ± 2.2^b^	+

a. Average of 18–21 plants.

b. Average of at least three fruits.

c. ++, >10 seeds/fruit; +, <5 seeds/fruit; -, absent.

### 3.2 Overexpression of *SlLIP1* in tomato fruits increases lipoylated protein and unbound LA levels

**Figure 2:**
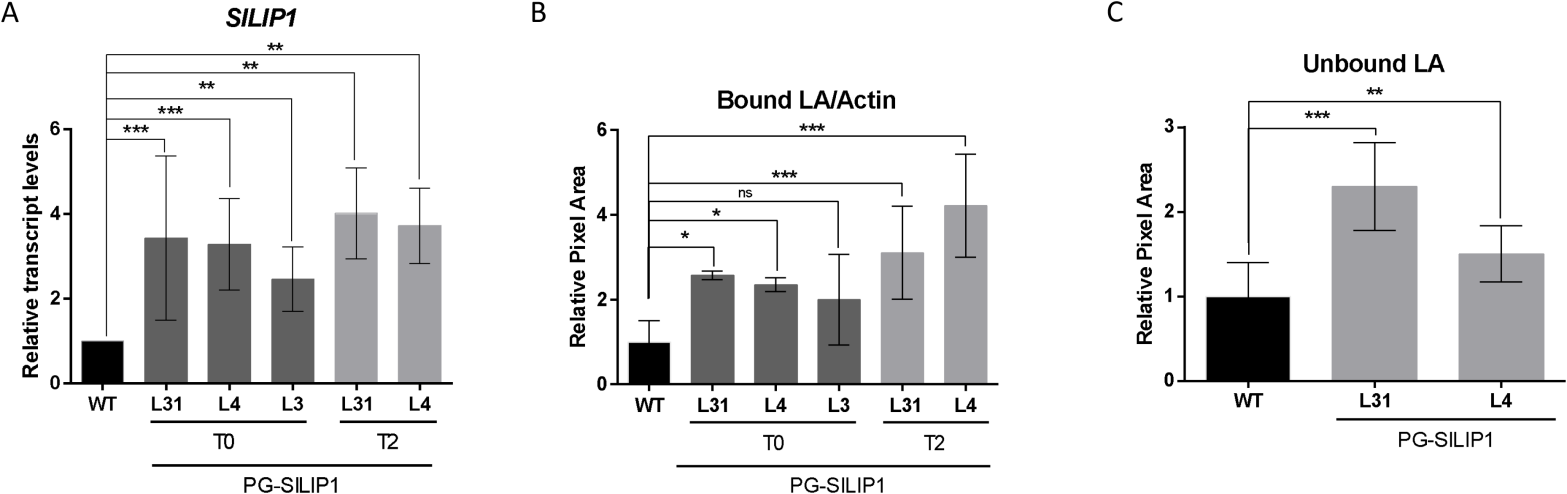
*SlLIP1* transcript levels and LA content in fruits of T0 and T2 PG-SlLIP1 transgenic tomato lines. **(A)** qRT-PCR analysis of *SlLIP1* was performed on three different fruits, each with two technical replicates (n=6 ± SD), normalized against *Actin7* and calibrated to WT levels. **(B)** Quantification of immunoblot signals of protein-bound LA using an anti-LA antibody, normalized to the signal of an anti-actin antibody in WT and PG-SlLIP1 transgenic fruits. All T0 and T2 samples were subjected to this analysis at least three times, whilst 5 replicates are considered for WT. Bars show means ± SD. **(C)** Detection of unbound LA in WT and T2 PG-SlLIP1 fruits (n=9 ± SD). p ≤ 0.05 (*), p ≤ 0.005 (**), and p ≤ 0.0005 (***) and not significant (n.s.).

The level of overexpression of *SlLIP1* was analyzed using qRT-PCR in mature red fruits from WT and transgenic PG-SlLIP1 lines across two different generations. Since the introduced *SlLIP1* is an endogenous gene in tomato, overexpression was defined as transcript levels higher than those in WT. Fruits from the transgenic lines PG-SlLIP1 L31, L4 and L3 at the T0 stage accumulated 2–3 fold higher transcript levels of *SlLIP1* than in WT. This pattern persisted into the T2 generation of L31 and L4 fruits (**Figure 2A**). Transcripts of introduced *SlLIP1* in fully-expanded leaves of all lines were absent, demonstrating that the PG promoter was not active in these organs (**Supplementary Figure 2**).

To determine whether this increase in *SlLIP1* transcript levels affected LA content, two experiments were conducted. First, lipoylated proteins were analyzed by immunoblotting, using an anti-LA antibody. The signal obtained was normalized to the signal of the anti-actin immunoblot, and the results were compared relative to WT, such that an increase in the LA/actin ratio suggests an increase in the lipoylation of target proteins (**Supplementary Figure 3**). In this context, fruits from T0 transgenic lines L31 and L4 showed up to ~2-fold higher lipoylated protein levels than WT. This rise was stably transmitted to the T2 generation (**Figure 2B**). The second approach measured free LA in extracts of hydrophilic antioxidants obtained from tomato fruits. To do so, a dot blot was performed using the same anti-LA antibody (**Supplementary Figure 4**). The results showed a significantly stronger signal in fruits from L31 and L4 at the T2 generation compared to WT fruits (**Figure 2C**). Overall, these results indicate that increasing *SlLIP1* levels in tomato fruits enhances bound and unbound LA content.

### 3.3 Transcript levels of LA biosynthesis and related genes are altered in transgenic tomato fruits with higher LA content

**Figure 3:**
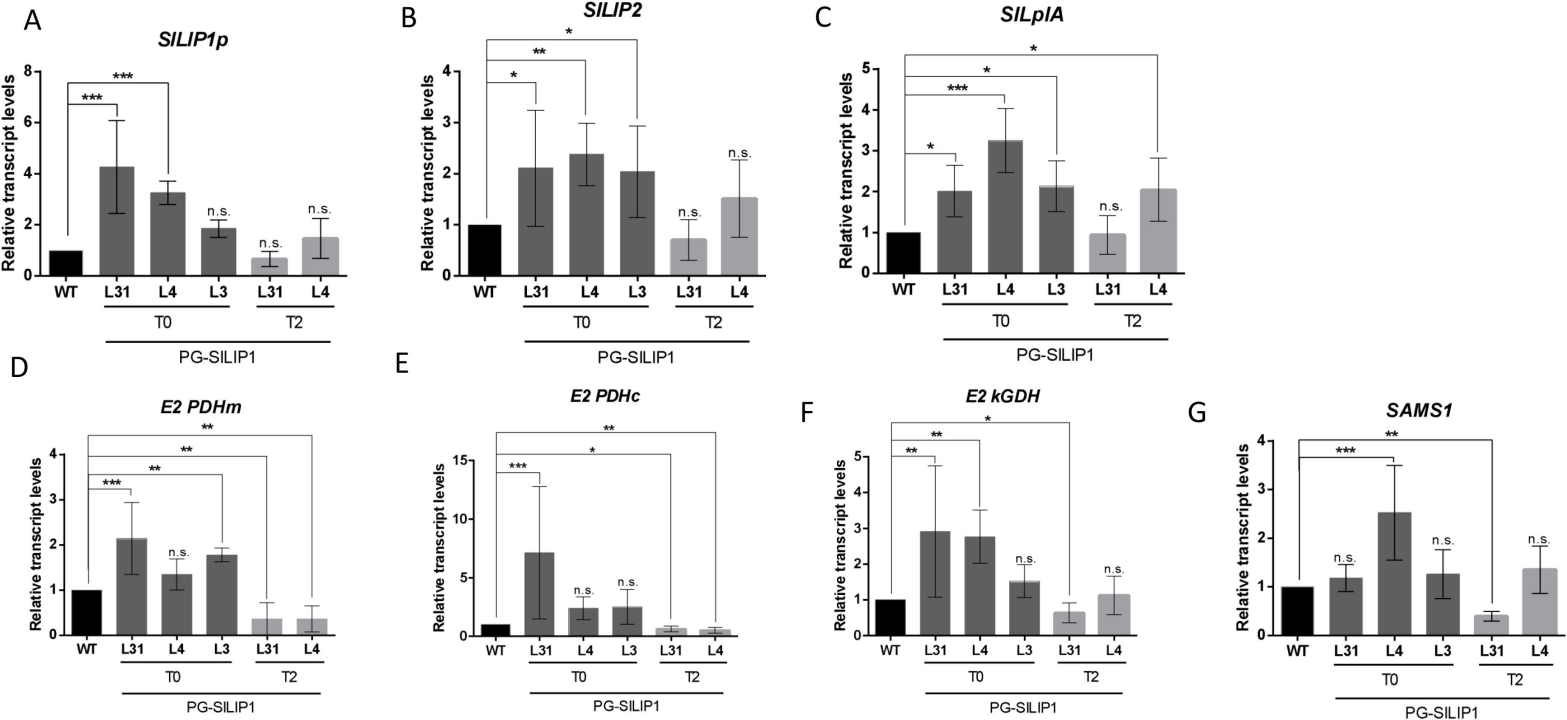
Relative transcript levels of genes involved in LA metabolism in fruits of T0 and T2 PG-SlLIP1 transgenic tomato lines. The analyzed genes are: **(A)** Plastid lipoyl synthase (*SlLIP1p*): an isoform of lipoyl synthase that contains a signal peptide targeting to chloroplasts. **(B)** Octanoyl transferase (*SlLIP2*): participates in *de novo* LA biosynthesis. **(C)** Lipoate protein ligase (*SlLplA*): involved in the salvage pathway. **(D)** Mitochondrial E2 subunit of pyruvate dehydrogenase (*E2 PDHm*). **(E)** Plastidial E2 subunit of pyruvate dehydrogenase (*E2 PDHc*). **(F)** E2 subunit of alpha-keto glutarate dehydrogenase (*E2 kGDH*). **(G)** SAM synthetase (*SAMS1*): involved in SAM biosynthesis. Transcript levels were normalized against *Actin7* and calibrated to WT levels. n=6 (T0 and T2) or 12 (WT) ± SD. p ≤ 0.05 (*), p ≤ 0.005 (**), p ≤ 0.0005 (***) and not significant (n.s.).

As an increase in *SlLIP1* transcript levels triggered a corresponding increase in bound and unbound LA content in fruits, the expression of various genes related to LA biosynthesis was analyzed to understand how transgenic fruits respond to the overexpression of *SlLIP1*. SlLIP1p (Solyc12g099700) is the plastidial lipoyl synthase isoform (Araya-Flores et al., 2020). *SlLIP1p* transcript levels in L31 and L4 at T0 were significantly higher than in WT fruits, while no differences were observed in L3 T0 or in both T2 lines (**Figure 3A**). On the other hand, the LA biosynthetic genes *SlLIP2* and *SlLplA* were more highly expressed in fruits from all the transgenic lines analyzed in the T0 generation (**Figures 3B, C**). Nevertheless, no differences were observed in the accumulation of *SlLIP2* transcripts in L31 and L4 at T2 (**Figure 3B**). In contrast, L4 at T2 showed higher transcript levels of *SlLplA* than WT and L31 fruits (**Figure 3C**).

Regarding the genes encoding lipoylated subunits, transcript levels of *E2 PDHm* (mitochondrial), *E2 PDHc* (plastidial) and *E2 kGDH* were evaluated. For *E2 PDHm*, transcript levels were higher in L31 and L3 at T0 compared to WT and L4. However, L31 and L4 at T2 showed lower transcript levels than WT (**Figure 3D**). In the case of *E2 PDHc*, L31 expressed this transcript more than WT, L4 and L3 fruits at T0, while L31 and L4 at T2 showed lower levels of transcript than WT (**Figure 3E**). For *E2 kGDH*, L31 and L4 at T0 showed higher transcript accumulation compared to WT, but at T2, L31 accumulated less of this transcript (**Figure 3F**).

Finally, during the enzymatic reaction of LIP1, SAM is consumed. Therefore, transcript levels of SAM synthetase 1 (*SAMS1*), the enzyme responsible for SAM synthesis (Zhang et al., 2020) were analyzed. L4 T0 fruits accumulated higher levels of this transcript, but this trend was not observed at T2. In contrast, L31 T2 fruits accumulated lower levels of *SAMS1* (**Figure 3G**). Taken together, these findings suggest that the fruit-specific overexpression of *SlLIP1* results in knock-on effects in transcript levels of lipoylation-related genes.

### 3.4 Primary metabolism is affected by the overexpression of *SlLIP1* in transgenic tomato fruits

**Figure 4:**
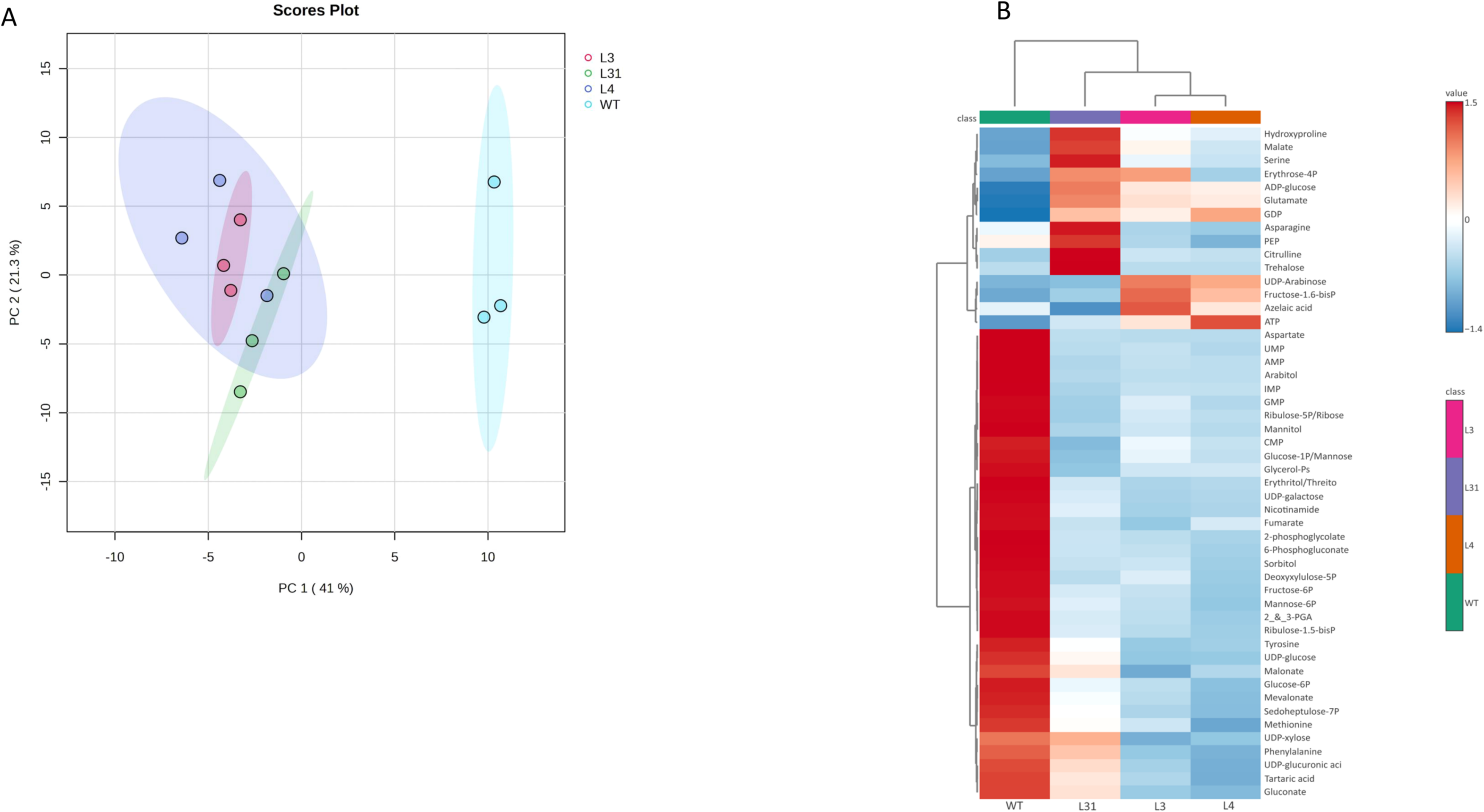
Targeted metabolomic analysis of fruits of T0 PG-SlLIP1 transgenic tomato lines. **(A)** Principal component analysis (PCA) was performed using detected metabolites as the predictor variable and genotype as the response variable. **(B)** Heatmap analysis of the top 50 significantly-altered metabolites identified by ANOVA. Columns represent the average of three biological replicates for each genotype (WT and PG-SlLIP1 lines L31, L4 and L3). The distance measure for clustering was Euclidean, and the clustering algorithm used was Ward.

To understand how the overexpression of *SlLIP1* and the increase in LA content modified the metabolite profiles in the PG-SlLIP1 tomato lines, a targeted metabolomic analysis of polar metabolites was performed using LC-MS/MS. A total of 99 metabolites were identified in mature red fruits, consisting of 10 sugars, 27 amino acids and their derivatives, and 62 organic acids and phosphorylated compounds. The data were evaluated through an unsupervised multivariate statistical analysis using the genotype as the response variable and metabolites as the predictor variable. Principal components analysis (PCA) of metabolites from transgenic PG-SlLIP1 (L31, L4 and L3) and WT tomato fruits explained 41% of the variance for PC1 and 21.3% for PC2. The projection clearly separated the transgenic and WT groups, with the three transgenic lines clustering together on the negative side of the plot (**Figure 4A**).

Next, the top 50 metabolites identified by ANOVA as being significantly-altered were clustered and visualized with a heatmap (**Figure 4B**). The three transgenic PG-SlLIP1 lines grouped together, distinct from WT. Thirty-five metabolites accumulated more in WT fruits than in transgenic lines, representing 70% of the metabolites identified. The remaining 15 metabolites accumulated more in the transgenic lines than in WT fruits, with four metabolites (asparagine, PEP, citrulline and trehalose) showing higher levels in L31 only. L4 and L3 fruits were more similar to each other, with four metabolites showing different patterns compared to L31 and WT fruits (UDP-arabinose, fructose 1,6-biphosphate, azelaic acid and ATP). Three metabolites (ADP-glucose, glutamate and GDP) accumulated more in all three transgenic lines than in WT. Erythrose 4-phosphate showed similar levels in L31 and L3 fruits.

**Figure 5:**
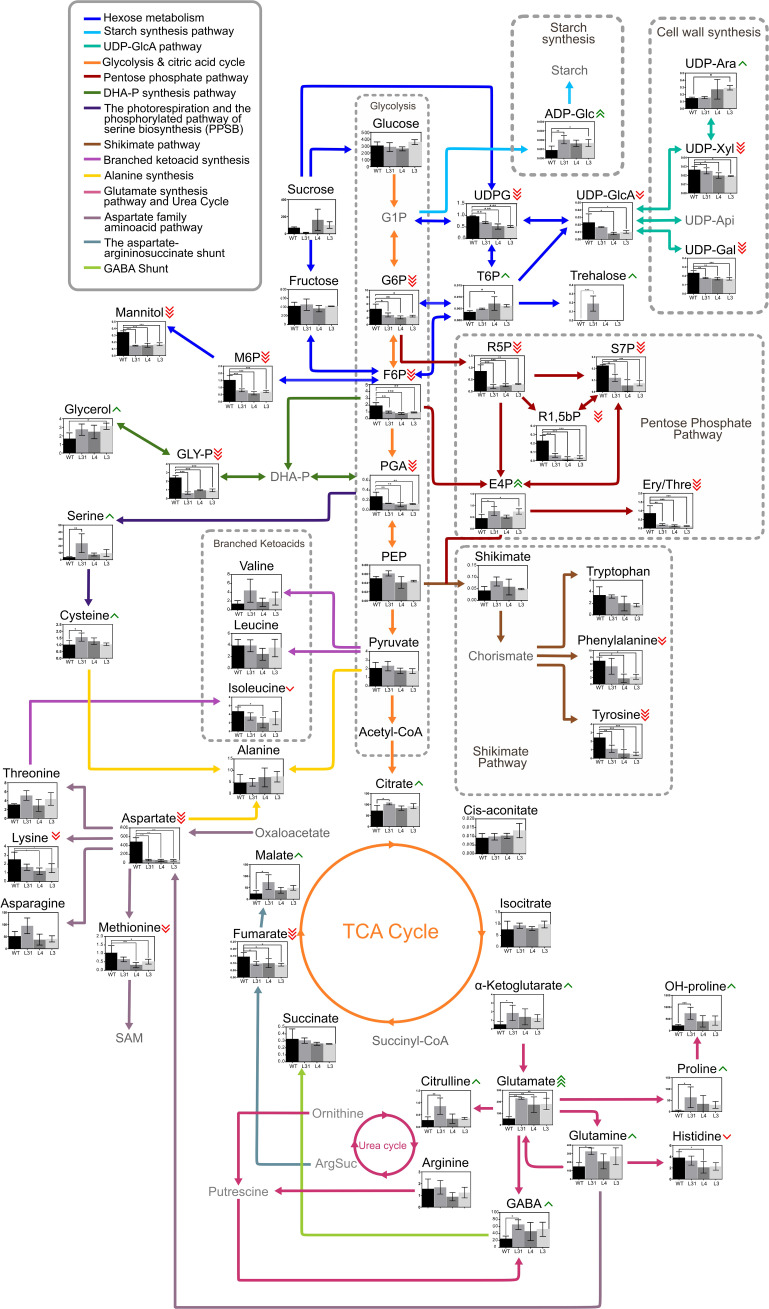
Metabolic comparison of fruits of PG-SlLIP1 transgenic and WT tomato lines. The graphical representation illustrates the variance in the metabolic profile of each transgenic fruit (n=3 ± SD) compared to WT (n=3 ± SD). Green arrows beside metabolite names indicate an increase in accumulation in transgenic fruits compared to WT, while red arrows indicate a decrease, according to the t-test with significance set at p ≤ 0.05 (*), (**) and (***). Gray metabolites were not measured but are included in the scheme to facilitate understanding. G1P, Glucose 1-phosphate; UDPG, UDP-glucose; UDP-GlcA, UDP-glucuronic acid; UDP-Ara, UDP-arabinose; UDP-Xyl, UDP-xylose, UDP-Api, UDP-apiose; UDP-Gal, UDP-galactose; ADP-Glc, ADP-glucose; G6P, Glucose 6-phosphate; T6P, Trehalose 6-phosphate; F6P, Fructose 6-phosphate; M6P, Mannose 6-phosphate; R5P, Ribulose 5-phosphate; S7P, Sedoheptulose 7-phosphate; R1,5-bP, Ribulose 1,5-biphosphate; E4P, Erythrose 4-phosphate; Ery/Thre, Erythritol/Threitol; PGA, 2–3 phosphoglyceric acid; DHA-P, Dihydroxyacetone phosphate; GLY-P, Glycerol phosphate; PEP, Phosphoenolpyruvate; SAM, S-adenosyl methionine; GABA, gamma-aminobutyric acid; OH-proline, hydroxyproline; ArgSuc, Argininosuccinate.

The changes in metabolite accumulation across the different transgenic lines provide insights into the metabolic pathways that may be altered due to the overexpression of *SlLIP1*. To explore this, a pathway enrichment analysis was performed by comparing metabolites of each transgenic line with WT (**Table 2**). The pentose phosphate pathway, starch and sucrose metabolism, fructose and mannose metabolism, alanine, aspartate and glutamate metabolism, and amino sugar and nucleotide sugar metabolism were the five metabolic pathways that were most represented in the three transgenic lines compared to WT fruits. The *p*-values from the enrichment analysis were statistically significant in all cases. This information was used to create a map illustrating the effect of *SlLIP1* overexpression in tomato fruits compared with WT (**Figure 5**).

**Table 2:** Metabolic pathways showing statistical differences in pathway enrichment analysis comparing WT to PG-SlLIP1 transgenic fruit metabolites.

Pathway Name	Match Status			*p*		
	L3	L4	L31	L3	L4	L31
Pentose phosphate pathway	6 of 19	6 of 19	6 of 19	4.92e-5	1.2e-03	1.2e-04
Starch and sucrose metabolism	9 of 22	9 of 22	10 of 22	6.83e-4	0.014	3.1e-04
Fructose and mannose metabolism	6 of 18	6 of 18	6 of 18	1.50e-4	1.2e-03	0.005
Alanine, aspartate and glutamate metabolism	9 of 22	9 of 22	9 of 22	0.007	0.038	0.007
Amino sugar and nucleotide sugar metabolism	14 of 52	14 of 52	14 of 52	5.29e-4	0.003	0.02

The main differences were observed in the pentose phosphate pathway, where four metabolites (ribulose 5-phosphate, ribulose 1,5-biphosphate, sedoheptulose 7-phosphate and erythritol/threitol) accumulated more in WT fruits, while erythrose 4-phosphate was more abundant in L3 fruits than in WT. Conversely, UDP-glucose, glucose 6-phosphate, and fructose 6-phosphate, related to starch and sucrose metabolism, along with metabolites related to amino sugar and nucleotide sugar metabolism (e.g., UDP-glucose, UDP-glucuronic acid, mannose 6-phosphate, UDP-xylose, fructose 6-phosphate, and UDP-galactose) accumulated more in WT fruits than in transgenic fruits (**Figure 5**). In contrast, trehalose 6-phosphate was more abundant in L4 fruits than WT, similar to ADP-glucose and UDP-arabinose. Regarding fructose and mannose metabolism, sorbitol, mannose 6-phosphate, and fructose 6-phosphate accumulated more in WT fruits, while fructose 1,5-biphosphate experienced the opposite. In alanine, aspartate and glutamate metabolism, aspartate and fumarate were more represented in WT fruits, whereas asparagine, glutamate (L31, L4 and L3), glutamine (L31), and GABA and proline (L31) were more abundant in transgenic lines than WT fruits (**Figure 5**). Therefore, these data highlight the central role of lipoylation in both primary and secondary metabolic pathways, such that overlipoylation results in a series of changes in polar metabolites.

## 4 Discussion

Lipoyl synthase is the enzyme that catalyzes the final step in LA biosynthesis by adding the two sulfur atoms to four octanoylated targets, PDH, kGDH, BCDH and GDC. Consistently, the effects of manipulating the expression of this enzyme throughout the whole plant are expected to be severe. Indeed, Ewald et al. (2014a) and Ewald et al. (2014) demonstrated that interruption of LIP2, LplA, LIP1p and LIP2p through T-DNA-mediated insertion in *Arabidopsis* resulted in a lethal phenotype when the T-DNA insertion was homozygous. This suggests that manipulating LA metabolism hinders normal plant growth. In this study, we used the PG promoter to overexpress tomato lipoyl synthase (*SlLIP1*) specifically during the breaker and pink fruit stages (**Supplementary Figure 1B**), thereby avoiding potential negative effects on vegetative organs. The increase in *SlLIP1* expression driven by the PG promoter in T0 and T2 generations was approximately 2- to 3- fold higher than in WT plants (**Figure 2**), similar to the ranges described by Arias et al. (2021) using the same pCP vector system. Additionally, no additional *SlLIP1* transcripts were detected in fully-expanded leaves of transformed plants (**Supplementary Figure 2**). The fruit-specific promoter chosen for this study corresponds to *SlPG14* (Solyc10g080210.1.1); indeed, a detailed analysis of the expression pattern of this gene shows that it is fruit-specific (Ke et al., 2018), consistent with publicly-available data (bar.utoronto.ca/efp2/Tomato/Tomato_eFPBrowser2).

The greater levels of *SlLIP1* transcripts triggered concomitant rises in both bound and unbound LA (**Figure 2**). These increases in LA content were associated with modifications in the transcript levels of various genes involved in LA biosynthesis (**Figures 3A, B, C**). To the best of our knowledge, there is no existing evidence detailing how this feedback mechanism operates between LIP1 and other genes in the LA biosynthesis pathway. However, in this study, we propose that elevated SlLIP1 activity results in depletion of octanoylated proteins which act as reaction substrates for lipoylation, leading to a positive feedback signal to upregulate SlLIP2 activity. This upregulation would enhance the transfer of octanoyl groups from acyl carrier protein (ACP) to target proteins, thus providing more potential substrates for SlLIP1. In the case of SlLplA, an enzyme associated with the salvage pathway that directly adds exogenous octanoyl or lipoyl groups onto target domains (Ewald et al., 2014a), we observed increased transcript levels in all three transgenic lines (**Figure 3C**). Therefore, we suggest a similar mechanism to that of SlLIP2, where substrate depletion caused by SlLIP1 activity upregulates SlLplA transcription through positive feedback, facilitating the uptake of free octanoyl and/or lipoyl from the medium and its transfer to target proteins.

Regarding the target proteins, we observed a significant increase in the transcript levels of mitochondrial and plastidial *E2-PDH*, as well as *E2 kGDH*, in some transgenic lines in the T0 generation. However, there were no differences observed in the T2 generation (**Figures 3D–F**). This is noteworthy, as these subunits must be lipoylated for them to be active (Douce et al., 2001; Mooney et al., 2002; Perham, 2000). In this context, there may be a positive feedback loop between LA biosynthesis and the transcription of genes encoding these target proteins. A similar phenomenon was observed in hepatocytes supplemented with LA, where researchers found an increase in mitochondrial PDH activity due to inhibition of its antagonist, pyruvate dehydrogenase kinase (PDK), through an unknown mechanism (Korotchkina et al., 2004). In contrast to the T0 lines, in the T2 generation the transcript levels of *E2-PDHm*, *E2-PDHc* and *E2 kGDH* were generally lower than in WT fruits. Along these lines, it has been demonstrated that there is an unlipoylated pool of E2 subunits of PDH and kGDH proteins in normal conditions in *Arabidopsis* (Ewald et al., 2014a). Therefore, we suggest that in the T2 generation, due to the stable overexpression of *SlLIP1* (**Figure 2A**) and the lower levels of transcripts of the genes encoding these lipoylated targets (**Figures 3D–F**; *E2-PDHm*, *E2-PDHc* and *E2 kGDH*), that the pool of unlipoylated E2 subunits has been reduced in the transgenic fruits. Indeed, and in support of this hypothesis, the immunoblot analysis shows that there are overall higher levels of bound LA in PG-SlLIP1 tomatoes compared to untransformed controls (**Figure 2B**).

Using targeted metabolome analysis of transgenic compared to WT fruits, we observed that the three PG-SlLIP1 lines were similar to each other but distinct from WT in terms of polar metabolite accumulation (**Figure 4**). Based on this data, we were able to identify which metabolic pathways were most affected by the overexpression of *SlLIP1* (**Table 2**) and subsequently created a diagram illustrating central metabolism and the metabolite accumulation in each genotype (**Figure 5**). Regarding starch and sucrose metabolism, glucose 6-phosphate (G6P) and UDP-glucose (UDPG) are two essential compounds in central metabolism within fruits. G6P belongs to the hexose pool and serves as a precursor for the pentose phosphate pathway, providing reducing power within the cells (Jiang et al., 2022). As for UDPG, it plays a key role in sucrose synthesis in source organs, as well as in the formation of cellulose and callose in the apoplast (Kleczkowski et al., 2010). Additionally, it is involved in the synthesis of hemicelluloses and pectins in the cell wall (Amor et al., 1995; Feingold and Barber, 1990; Gibeaut, 2000; Johansson et al., 2002; Kleczkowski, 1994; Reiter, 2008). A mutant of the *ugpA* gene in *Arabidopsis*, which encodes UDP-glucose pyrophosphorylase responsible for synthesizing UDPG from glucose 1-phosphate, exhibited 50% less seed production compared to WT plants (Meng et al., 2009). This finding is consistent with observations in PG-SlLIP1 lines, which showed reduced or absent seed production (**Table 1**). Metabolomic data indicated that G6P and UDPG levels were lower in PG-SlLIP1 fruits compared to WT. Additionally, trehalose 6-phosphate (T6P) accumulated more in PG-SlLIP1 L4 and tended to be higher in L31 and L3 fruits (**Figure 5**). G6P and UDPG serve as precursors to T6P (Kerbler et al., 2023), which is subsequently dephosphorylated by T6P phosphatase to produce trehalose. Meanwhile, fatty acid synthesis is regulated by the WRI1 transcription factor (Zhai et al., 2018). When phosphorylated by SnRK1 kinase, WRI1 cannot translocate to the nucleus. T6P can bind to the SnRK1 kinase catalytic subunit, inhibiting its activation, thus allowing WRI1 to enter the nucleus and activate fatty acid synthesis (Zhai et al., 2018). Since LA is derived from fatty acid biosynthesis (octanoylated-ACP) when synthesized *de novo*, we propose that the observed increase in LA is, at least in part, being sustained by the elevated T6P levels.

As previously mentioned, SAM is a key molecule in LIP1 activity, as one molecule of SAM is consumed for every two sulfur atoms inserted into octanoylated proteins (Harmer et al., 2014). The biosynthesis of SAM is catalyzed by SAMS, with methionine serving as a precursor. Previous studies have mutated one of the four *SAMS* genes in *Arabidopsis*, leading to an increase in methionine levels. Consequently, mutant plants exhibit a severe growth defect due to reduced SAM levels, which are essential for methylation reactions (Goto et al., 2002; Jander and Joshi, 2010; Shen et al., 2002). Lysine, or a related metabolite, regulate SAMS at the transcriptional level and modulate its enzymatic activity (Hacham et al., 2007). Inhibition of SAMS reduces SAM biosynthesis and consequently leads to methionine accumulation, as SAM is a negative regulator of cystathionine gamma-synthase, which is involved in the initial step of methionine biosynthesis (Jander and Joshi, 2010). Our results showed a decrease in lysine levels in transgenic tomato fruits (**Figure 5**), suggesting that this may enable SAMS to become more active, thereby consuming more methionine to generate additional SAM for elevated LIP1 activity.

The metabolomic data showed an increase in glutamate levels in the three transgenic lines, along with a statistically significant increase in glutamine and GABA levels in L31, and a trend towards increased levels in L4 and L3 (**Figure 5**). These results suggest an activation of the “GABA shunt”, which bypasses two steps of the TCA cycle, specifically succinyl-CoA ligase and kGDH (Bouché and Fromm, 2004), the latter of which is a lipoylated target. This pathway involves three key enzymes: glutamate decarboxylase (GAD), GABA transaminase (GABA-T) and succinic semialdehyde dehydrogenase (SSADH) (Michaeli and Fromm, 2015). The metabolomic results indicate a potential activation of the shunt, possibly due to deregulation of kGDH as a result of higher LIP1 activity. Another possible explanation is that the transgenic fruits were responding to oxidative stress, which could have triggered an increase in Ca^2+^ levels, subsequently activating the GABA shunt, as GAD is regulated by intracellular Ca^2+^. Notably, changes in intracellular Ca^2+^ levels are known to be related to ROS response and signaling in plants (Ansari et al., 2021). Indeed, high proline content is an indicator of the stress response (Kavi Kishor and Sreenivasulu, 2014). This was corroborated by our metabolomic data, where L31 exhibited statistically-significantly higher proline levels compared to WT (**Figure 5**). Similar findings were reported by (Ewald et al., 2014a), where *LplA* RNAi plants showed elevated proline and GABA levels, indicating metabolic stress due to decreased E2-PDH lipoylation in leaves and roots in *Arabidopsis*. In contrast, our results showed higher levels of lipoylated proteins, suggesting that both greater and lower lipoylation may induce metabolic stress in plant cells. Nevertheless, the metabolomic analysis also demonstrates the uniformity of the mature red fruits that were chosen for these assays, as the levels of Glc and Fru [sugars known to accumulate in mature red fruits, **Figure 5**; Carrari and Fernie (2006)] were identical between transgenic and WT fruits, consistent with our expression data showing that transcript levels of *SlPG14* and *SlNAP2*, genes whose expression correlates positively with fruit development (Ke et al., 2018; Kou et al., 2016) are similar between transgenic and WT lines (**Supplementary Figures 1A, B**).

In conclusion, the use of the PG promoter has made it possible to obtain stably transformed tomato plants whose plant phenotypes did not differ significantly from WT. Using this promoter, *LIP1* was overexpressed in mature fruits, but not in leaves. Overexpression of *SlLIP1* was observed to not only increase bound and unbound levels of LA but additionally it raised the expression of many other genes associated with LA metabolism. Higher *SlLIP1* expression was also observed to have effects on metabolite abundance associated with various metabolic routes, among which we highlight general reductions in metabolites participating in glycolysis and the pentose phosphate pathway, and a marked increase in glutamate and some of its downstream products such as glutamine and GABA, pointing towards activation of the GABA shunt. The applications of this work mean that SlLIP1 is a potential target for increasing LA levels in antioxidant-poor crops in an organ-specific manner, without incurring substantial physiological costs on their performance.

## Data Availability

The original contributions presented in the study are included in the article/**Supplementary Material**, further inquiries can be directed to the corresponding author.
