# Supplementary material for: Fruit-specific overexpression of lipoyl synthase increases both bound and unbound lipoic acid and alters the metabolome of tomato fruits
Source: Front Plant Sci. 2025 May 19;16:1545011. doi: 10.3389/fpls.2025.1545011 (PMC12127388; doi:10.3389/fpls.2025.1545011)
Supplement: Supplementary file 1 [file Presentation1.pptx]

## Slide 1
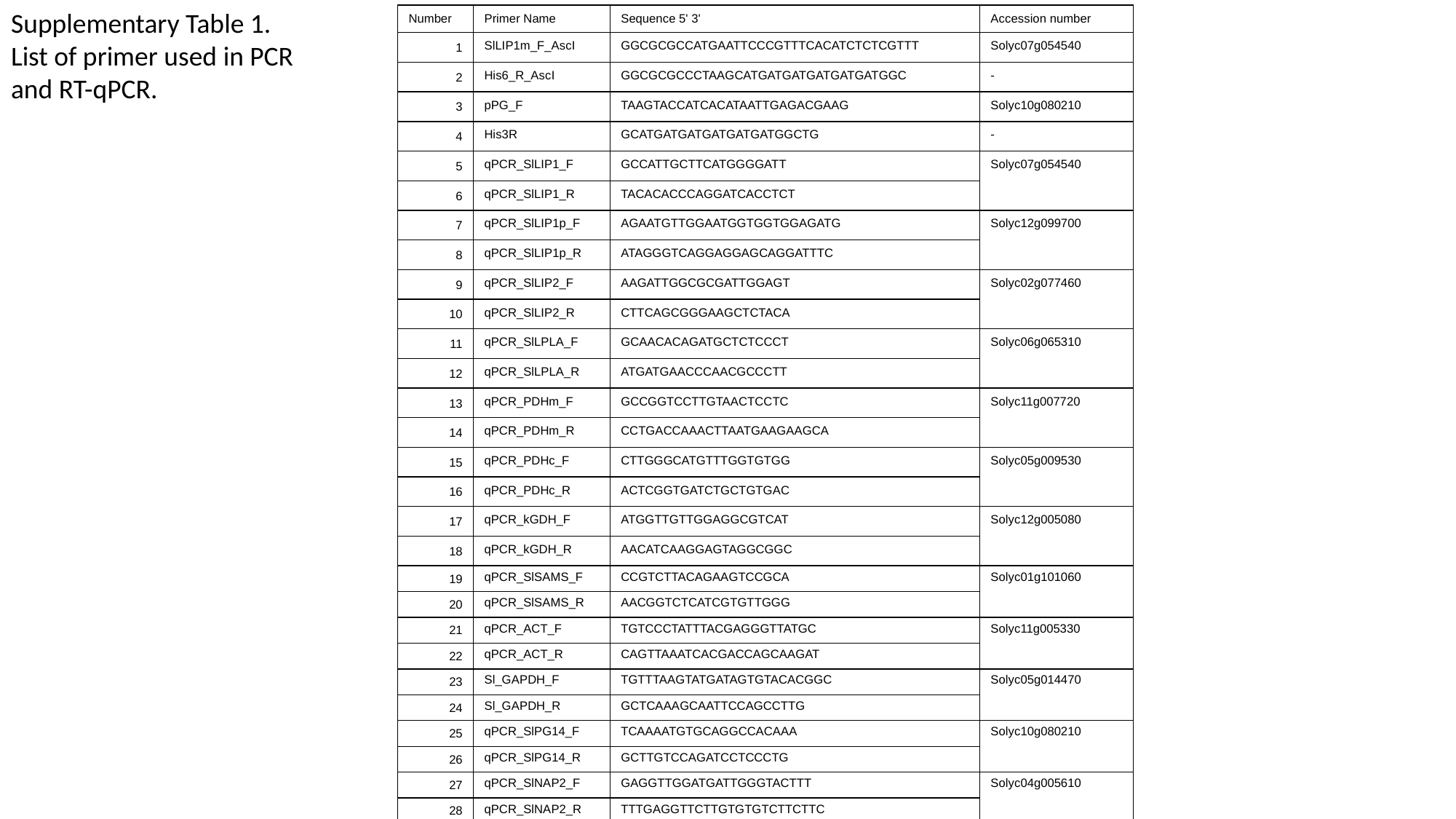

Supplementary Table 1.
List of primer used in PCR
and RT-qPCR.
| Number | Primer Name | Sequence 5' 3' | Accession number |
| --- | --- | --- | --- |
| 1 | SlLIP1m\_F\_AscI | GGCGCGCCATGAATTCCCGTTTCACATCTCTCGTTT | Solyc07g054540 |
| 2 | His6\_R\_AscI | GGCGCGCCCTAAGCATGATGATGATGATGATGGC | - |
| 3 | pPG\_F | TAAGTACCATCACATAATTGAGACGAAG | Solyc10g080210 |
| 4 | His3R | GCATGATGATGATGATGATGGCTG | - |
| 5 | qPCR\_SlLIP1\_F | GCCATTGCTTCATGGGGATT | Solyc07g054540 |
| 6 | qPCR\_SlLIP1\_R | TACACACCCAGGATCACCTCT | |
| 7 | qPCR\_SlLIP1p\_F | AGAATGTTGGAATGGTGGTGGAGATG | Solyc12g099700 |
| 8 | qPCR\_SlLIP1p\_R | ATAGGGTCAGGAGGAGCAGGATTTC | |
| 9 | qPCR\_SlLIP2\_F | AAGATTGGCGCGATTGGAGT | Solyc02g077460 |
| 10 | qPCR\_SlLIP2\_R | CTTCAGCGGGAAGCTCTACA | |
| 11 | qPCR\_SlLPLA\_F | GCAACACAGATGCTCTCCCT | Solyc06g065310 |
| 12 | qPCR\_SlLPLA\_R | ATGATGAACCCAACGCCCTT | |
| 13 | qPCR\_PDHm\_F | GCCGGTCCTTGTAACTCCTC | Solyc11g007720 |
| 14 | qPCR\_PDHm\_R | CCTGACCAAACTTAATGAAGAAGCA | |
| 15 | qPCR\_PDHc\_F | CTTGGGCATGTTTGGTGTGG | Solyc05g009530 |
| 16 | qPCR\_PDHc\_R | ACTCGGTGATCTGCTGTGAC | |
| 17 | qPCR\_kGDH\_F | ATGGTTGTTGGAGGCGTCAT | Solyc12g005080 |
| 18 | qPCR\_kGDH\_R | AACATCAAGGAGTAGGCGGC | |
| 19 | qPCR\_SlSAMS\_F | CCGTCTTACAGAAGTCCGCA | Solyc01g101060 |
| 20 | qPCR\_SlSAMS\_R | AACGGTCTCATCGTGTTGGG | |
| 21 | qPCR\_ACT\_F | TGTCCCTATTTACGAGGGTTATGC | Solyc11g005330 |
| 22 | qPCR\_ACT\_R | CAGTTAAATCACGACCAGCAAGAT | |
| 23 | Sl\_GAPDH\_F | TGTTTAAGTATGATAGTGTACACGGC | Solyc05g014470 |
| 24 | Sl\_GAPDH\_R | GCTCAAAGCAATTCCAGCCTTG | |
| 25 | qPCR\_SlPG14\_F | TCAAAATGTGCAGGCCACAAA | Solyc10g080210 |
| 26 | qPCR\_SlPG14\_R | GCTTGTCCAGATCCTCCCTG | |
| 27 | qPCR\_SlNAP2\_F | GAGGTTGGATGATTGGGTACTTT | Solyc04g005610 |
| 28 | qPCR\_SlNAP2\_R | TTTGAGGTTCTTGTGTGTCTTCTTC | |

## Slide 2
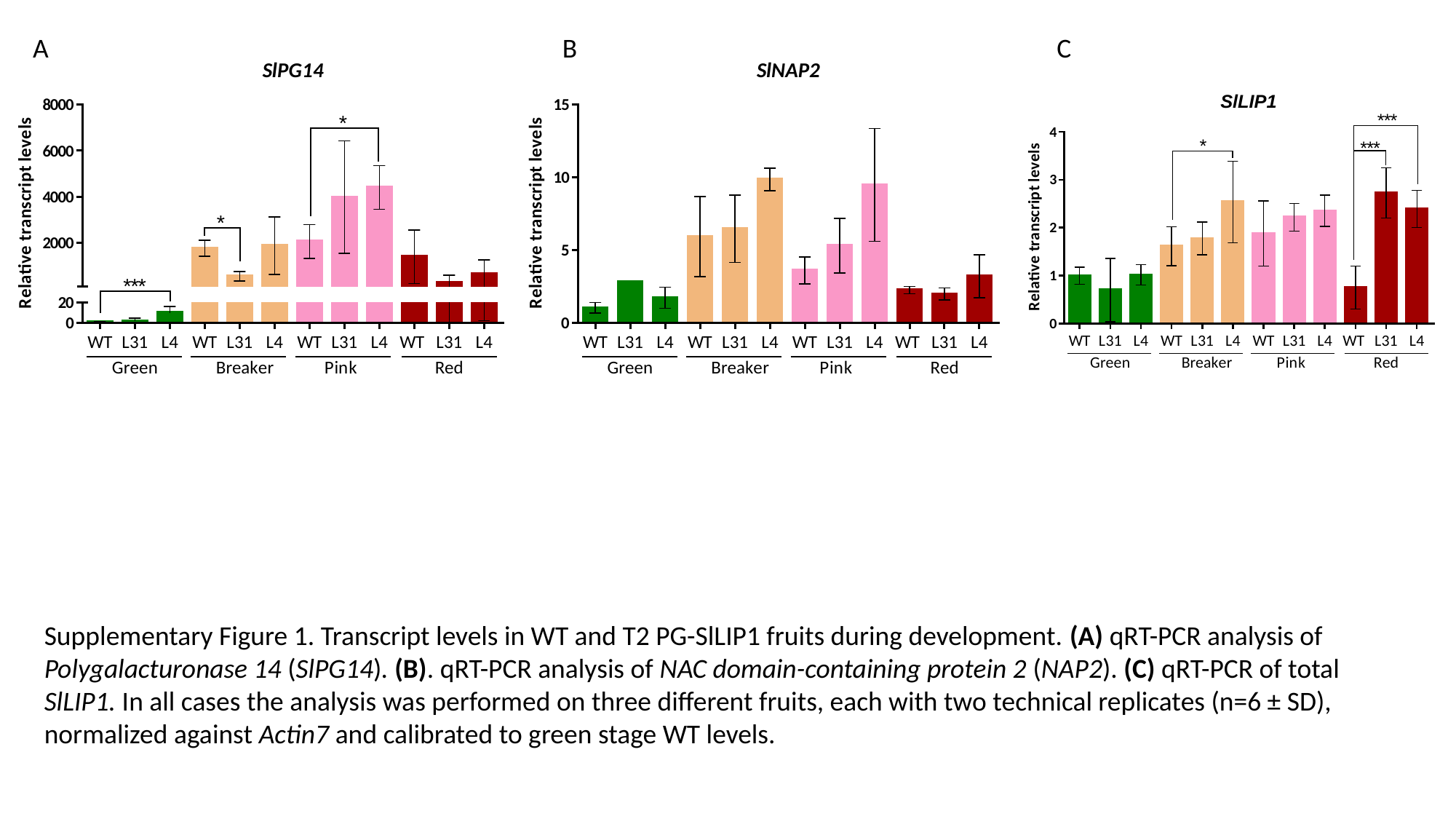

C
A
B
Supplementary Figure 1. Transcript levels in WT and T2 PG-SlLIP1 fruits during development. (A) qRT-PCR analysis of Polygalacturonase 14 (SlPG14). (B). qRT-PCR analysis of NAC domain-containing protein 2 (NAP2). (C) qRT-PCR of total SlLIP1. In all cases the analysis was performed on three different fruits, each with two technical replicates (n=6 ± SD), normalized against Actin7 and calibrated to green stage WT levels.

## Slide 3
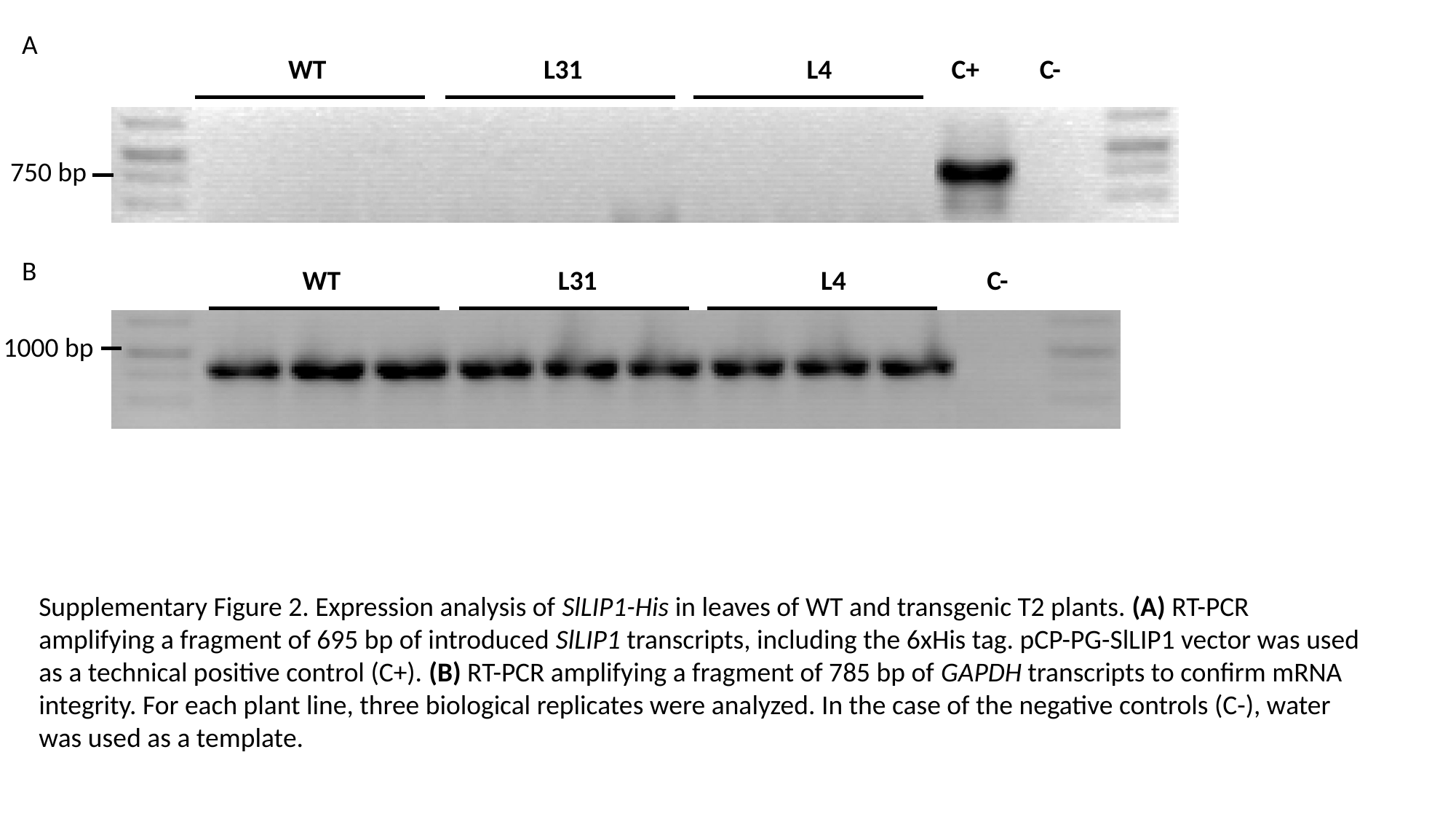

A
WT
L31
L4
C+
C-
750 bp
B
C-
WT
L31
L4
1000 bp
Supplementary Figure 2. Expression analysis of SlLIP1-His in leaves of WT and transgenic T2 plants. (A) RT-PCR amplifying a fragment of 695 bp of introduced SlLIP1 transcripts, including the 6xHis tag. pCP-PG-SlLIP1 vector was used as a technical positive control (C+). (B) RT-PCR amplifying a fragment of 785 bp of GAPDH transcripts to confirm mRNA integrity. For each plant line, three biological replicates were analyzed. In the case of the negative controls (C-), water was used as a template.

## Slide 4
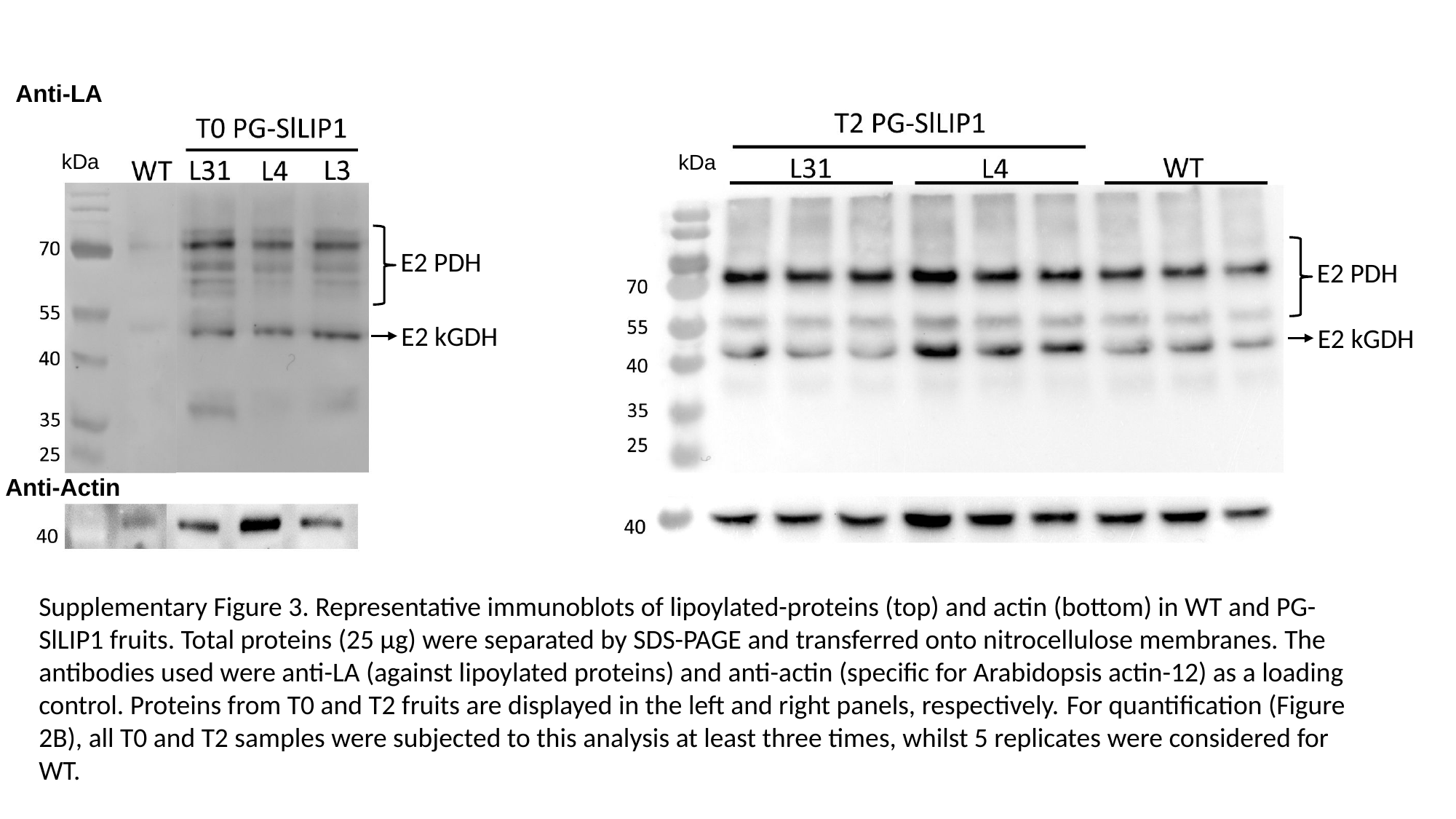

Anti-LA
kDa
kDa
E2 PDH
E2 PDH
E2 kGDH
E2 kGDH
Anti-Actin
Supplementary Figure 3. Representative immunoblots of lipoylated-proteins (top) and actin (bottom) in WT and PG-SlLIP1 fruits. Total proteins (25 μg) were separated by SDS-PAGE and transferred onto nitrocellulose membranes. The antibodies used were anti-LA (against lipoylated proteins) and anti-actin (specific for Arabidopsis actin-12) as a loading control. Proteins from T0 and T2 fruits are displayed in the left and right panels, respectively. For quantification (Figure 2B), all T0 and T2 samples were subjected to this analysis at least three times, whilst 5 replicates were considered for WT.

## Slide 5
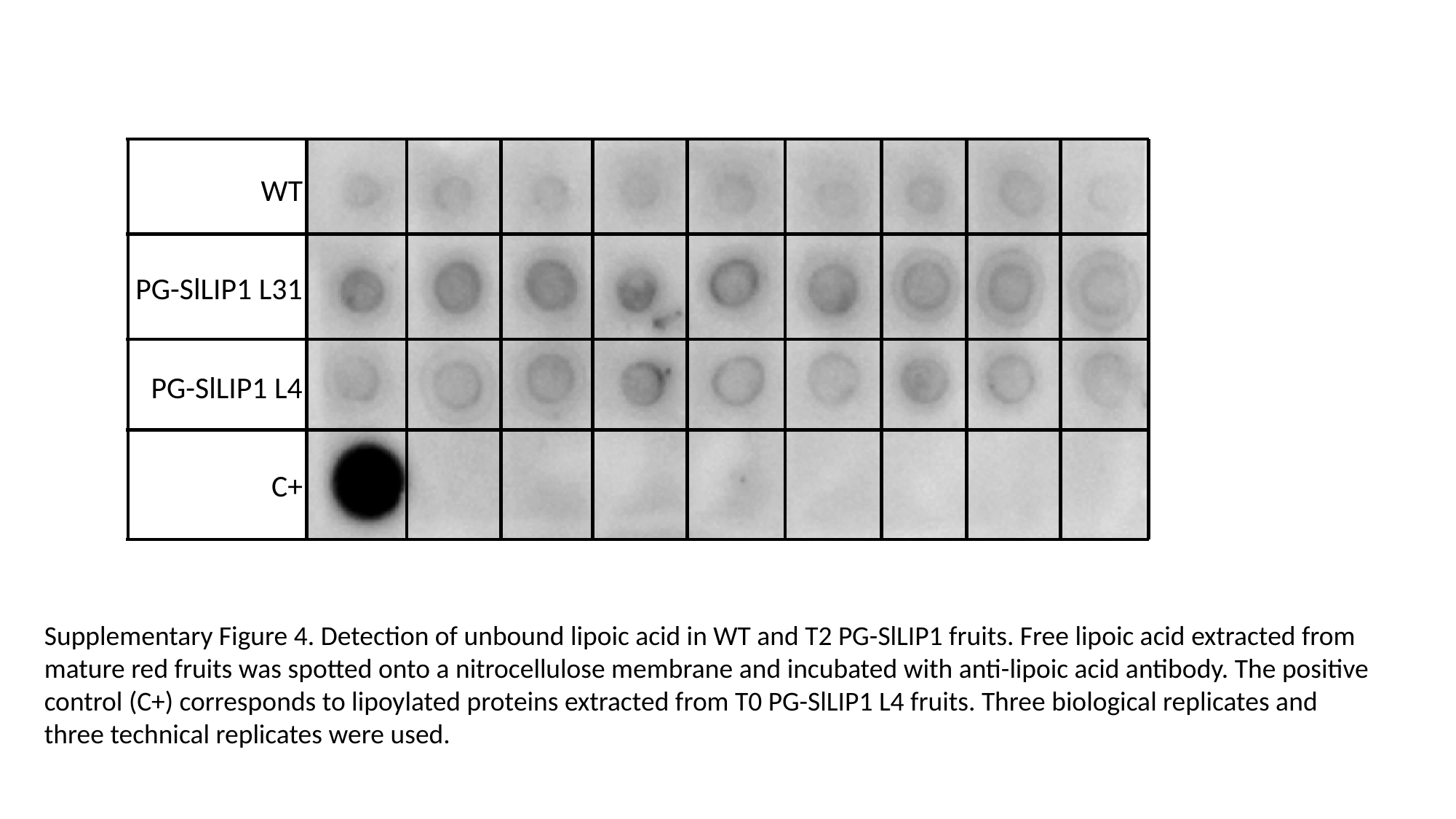

WT
PG-SlLIP1 L31
PG-SlLIP1 L4
C+
Supplementary Figure 4. Detection of unbound lipoic acid in WT and T2 PG-SlLIP1 fruits. Free lipoic acid extracted from mature red fruits was spotted onto a nitrocellulose membrane and incubated with anti-lipoic acid antibody. The positive control (C+) corresponds to lipoylated proteins extracted from T0 PG-SlLIP1 L4 fruits. Three biological replicates and three technical replicates were used.
